# Low carbohydrate high fat ketogenic diets on the exercise crossover point and glucose homeostasis

**DOI:** 10.3389/fphys.2023.1150265

**Published:** 2023-03-28

**Authors:** T. D. Noakes, P. J. Prins, J. S. Volek, D. P. D’Agostino, A. P. Koutnik

**Affiliations:** ^1^ Department of Medical and Wellness Science, Cape Peninsula University of Technology, Cape Town, South Africa; ^2^ Department of Exercise Science, Grove City College, Grove City, PA, United States; ^3^ Department of Human Sciences, The Ohio State University, Columbus, OH, United States; ^4^ Department of Molecular Pharmacology and Physiology, University of South Florida, Tampa, FL, United States; ^5^ Human Healthspan, Resilience and Performance, Institute of Human and Machine Cognition, Pensacola, FL, United States

**Keywords:** low carbohydrate, ketogenic diet (KD), crossover concept, exercise, fat, oxidation, prediabetes, continuous glucose monitor (CGM)

## Abstract

In exercise science, the crossover effect denotes that fat oxidation is the primary fuel at rest and during low-intensity exercise with a shift towards an increased reliance on carbohydrate oxidation at moderate to high exercise intensities. This model makes four predictions: First, >50% of energy comes from carbohydrate oxidation at ≥60% of maximum oxygen consumption (VO_2_max), termed the crossover point. Second, each individual has a maximum fat oxidation capacity (FATMAX) at an exercise intensity lower than the crossover point. FATMAX values are typically 0.3–0.6 g/min. Third, fat oxidation is minimized during exercise ≥85%VO_2_max, making carbohydrates the predominant energetic substrate during high-intensity exercise, especially at >85%VO_2_max. Fourth, high-carbohydrate low-fat (HCLF) diets will produce superior exercise performances *via* maximizing pre-exercise storage of this predominant exercise substrate. In a series of recent publications evaluating the metabolic and performance effects of low-carbohydrate high-fat (LCHF/ketogenic) diet adaptations during exercise of different intensities, we provide findings that challenge this model and these four predictions. First, we show that adaptation to the LCHF diet shifts the crossover point to a higher %VO_2_max (>80%VO_2_max) than previously reported. Second, substantially higher FATMAX values (>1.5 g/min) can be measured in athletes adapted to the LCHF diet. Third, endurance athletes exercising at >85%VO_2_max, whilst performing 6 × 800 m running intervals, measured the highest rates of fat oxidation yet reported in humans. Peak fat oxidation rates measured at 86.4 ± 6.2%VO_2_max were 1.58 ± 0.33 g/min with 30% of subjects achieving >1.85 g/min. These studies challenge the prevailing doctrine that carbohydrates are the predominant oxidized fuel during high-intensity exercise. We recently found that 30% of middle-aged competitive athletes presented with pre-diabetic glycemic values while on an HCLF diet, which was reversed on LCHF. We speculate that these rapid changes between diet, insulin, glucose homeostasis, and fat oxidation might be linked by diet-induced changes in mitochondrial function and insulin action. Together, we demonstrate evidence that challenges the current crossover concept and demonstrate evidence that a LCHF diet may also reverse features of pre-diabetes and future metabolic disease risk, demonstrating the impact of dietary choice has extended beyond physical performance even in athletic populations.

## Exercise crossover concept

**FIGURE 1:**
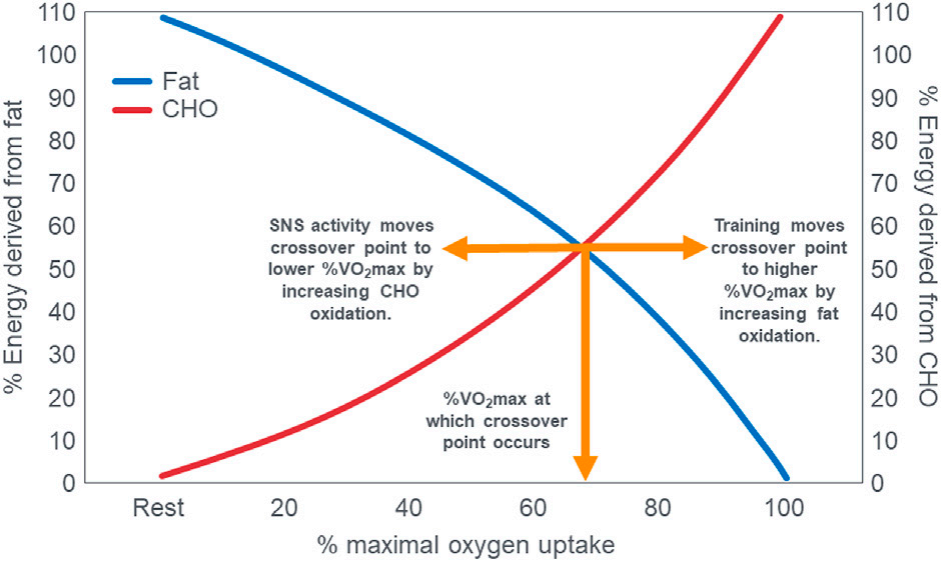
The relation between the relative contributions of fat and carbohydrate (CHO) utilization to overall energy expenditure as a function of exercise intensity. The point at which the two lines intersect illustrates the classic crossover concept. Reproduced and redrawn from (Brooks and Mercier, 1985; Kenney et al., 2021).

A time-honored physiological principle is that energy metabolism during exercise of increasing intensity changes from a predominance of energy derived from fat oxidation at lower exercise intensities to an increased reliance on carbohydrate oxidation at higher exercise intensities (Achten et al., 2002; Achten and Jeukendrup, 2003; Venables et al., 1985; Jeukendrup and Wallis, 2005; Randell et al., 2017; Frandsen et al., 2017). Ultimately at the highest exercise intensities, expressed as a percentage of maximal oxygen consumption (VO_2_max), carbohydrate becomes the exclusive or obligatory fuel. Above an exercise intensity of 85%VO_2_max, humans are no longer able to extract any energy from fat oxidation (Achten et al., 2002; Achten and Jeukendrup, 2003; Venables et al., 1985; Jeukendrup and Wallis, 2005; Randell et al., 2017; Frandsen et al., 2017; Vest et al., 2018; Brooks, 1997; Brooks, 1998; Brooks and Mercier, 1985; Brooks and Trimmer, 1985; Kolodziej and O'Halloran, 2021; Kenney et al., 2021). This concept is depicted graphically [Figure 1; (Brooks and Mercier, 1985; Kenney et al., 2021)] as the “crossover” point which is defined as “the power output at which energy from carbohydrates-derived fuels predominates over energy from lipids, with further increases in power eliciting a relative increment in carbohydrate utilization and a decrement in lipid oxidation” (Brooks and Mercier, 1985). The proposed explanations for this phenomenon are the following: “2. Lipid is the major fuel (approximately 60%) for non-contracting muscle and the body at rest. 3. Energy flux, as determined by exercise intensity, is the major factor determining the balance of substrate oxidation during exercise. Thus, moderate and greater exercise intensities increase contraction-induced muscle glycogenolysis and glycolysis, increase recruitment of fast-twitch muscle fibers, increase sympathetic nervous system (SNS) activity and downregulate mitochondrial fatty acid uptake. 4. Glycogen and glucose utilization scale exponentially to relative exercise power output with a greater gain in glycogen than in glucose use at high power. The relationship between free fatty acid (FFA) flux and power output is an inverted hyperbola. Consequently, at high power outputs, the role of lipid oxidation is diminished. 5. Factors such as endurance training, energy supply, as influenced by dietary manipulation, and prior exercise play secondary roles in determining the balance of substrate utilization during exercise” [(Brooks, 1997), p. 889]. In addition, “Prior endurance training results in muscular biochemical adaptations that enhance lipid oxidation as well as decrease the SNS response to given submaximal exercise stresses. These adaptations promote lipid oxidation during mild-to moderate-intensity exercise. In contrast, increases in exercise intensity are conceived to increase contraction-induced muscle glycogenolysis, alter the pattern of fiber type recruitment, and increase SNS activity” [(Brooks and Mercier, 1985), p. 2253]. Type 2 diabetes mellitus (T2DM) increases carbohydrate oxidation at low exercise intensities (Ghanassia et al., 2006) and elevates SNS activity (Huggett et al., 2003; Thackeray et al., 2012). According to this interpretation, the point at which this crossover from fat to carbohydrate oxidation occurs with increasing intensity is relatively fixed so that “factors such as endurance training, energy supply as influenced by dietary manipulation, and prior exercise play secondary role in determining the balance of substrate oxidation during exercise” [(Brooks, 1997), p.889].

**FIGURE 2:**
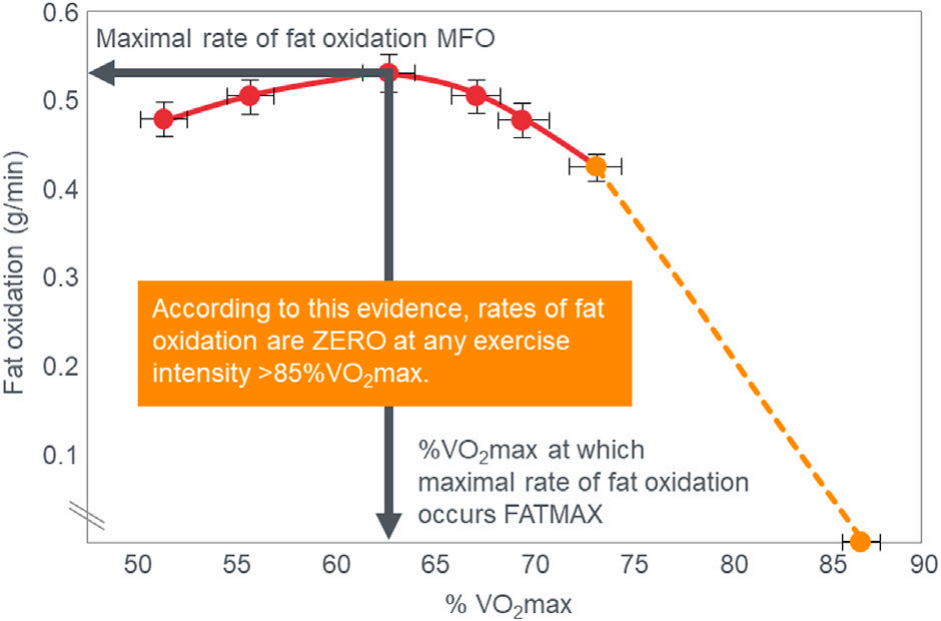
According to the crossover concept, rates of fat oxidation initially increase during exercise of low to moderate intensity reaching a maximal rate of fat oxidation (MFO) at between 60%–65%VO_2_max (FATMAX) before falling steeply as the exercise intensity increases further. The model predicts that the rate of fat oxidation reaches zero at exercise intensities greater than 85%VO_2_max. Redrawn from reference (Achten and Jeukendrup, 2003).

The crossover concept (Brooks, 1997; Brooks, 1998; Brooks and Mercier, 1985; Brooks and Trimmer, 1985; Kolodziej and O’Halloran, 2021; Kenney et al., 2021) has contributed to a commonly held belief that high-carbohydrate diets are ergogenic whereas high-fat diets are more likely to impair performance, especially during high-intensity or more prolonged exercise whilst sustaining higher intensities as it is believed that carbohydrate oxidation is required and preferred (Burke, 2015). Thus: “For most events at the Olympics (and others), carbohydrate is the primary fuel for anaerobic and aerobic metabolism” [(Hargreaves and Spriet, 2020), p.817] so that “Fat-derived ATP production is designed to provide a ‘helper fuel’ during exercise, with a maximum amount of energy at power outputs of ∼60–65%VO_2_max (Achten and Jeukendrup, 2003)” [ (Hargreaves and Spriet, 2020), p.820]. A logical prediction of the crossover concept is that each human, on average, has a maximal rate of fat oxidation that occurs at ∼65%VO_2_max (Figure 2) with some analyses suggesting maximal rate of fat oxidation may be achieved at slightly lower intensities [48%VO_2_max; (Venables et al., 1985)]. Figure 2 shows fat oxidation increases somewhat as the exercise intensity increases from 50 to ∼63%VO_2_max and fall steeply as exercise reaches higher intensities. Above an exercise intensity of approximately 85%VO_2_max, the rate of fat oxidation reaches zero. According to this model, peak rates of fat oxidation measured at exercise intensities below ∼61%VO_2_max vary from approximately 0.2–0.6 g/min in different populations (Randell et al., 2017; Maunder et al., 2018; Chrzanowski-Smith et al., 2020; Chrzanowski-Smith et al., 2021) although some individual values approaching 1 g/min have been measured in both male and female Ironman triathletes (Frandsen et al., 2017; Vest et al., 2018). A well-known limitation of estimating substrate oxidation from gas exchange is the underestimation of fat oxidation at exercise intensities >70% VO_2_max. During progressive exercise, high rates of glycolysis generate an accumulation of H^+^ in the contracting muscle that are transported to the extracellular fluid, which is buffered by [HCO_3_^−^]. This excess (non-oxidative) CO_2_ is excreted through hyperpnoea, elevating the VCO_2_. As a result, indirect calorimetry artifactually elevates respiratory exchange ratio (RER) thereby overestimating carbohydrate oxidation and underestimating fat oxidation during high-intensity exercise >70% VO_2_max. The extent to which these artifacts influence measurement of fat oxidation *via* indirect calorimetry in low-carbohydrate adapted athletes remains unclear as they would be expected to have lower rates of glycolysis and H^+^ generation. To the extent this occurs at some point during progressive exercise, it would underestimate the reported fat oxidation rates.

## Low-carbohydrate high-fat diets and the crossover point

### Substrate oxidation

In 2016 Volek et al. (Volek et al., 2016) reported much higher rates of fat oxidation in ultramarathon runners chronically adapted to an LCHF diet. Peak rates of fat oxidation were significantly higher in these runners than in a control group who followed the traditional high-carbohydrate low-fat (HCLF) diet but were otherwise matched for athletic caliber and VO_2_max [1.6 g/min vs. 0.7 g/min; Figure 2 in ref (Volek et al., 2016)]. Peak rates of fat oxidation also occurred at a higher %VO_2_max in the ultramarathoners eating the LCHF diet [70% vs. 55%; Figure 2 in reference (Volek et al., 2016)]. A reasonable conclusion might be that the higher rates of fat oxidation in ultramarathoners habituated to the LCHF diet is likely due to whole-body metabolic adaptions (Kolodziej and O'Halloran, 2021; Chrzanowski-Smith et al., 2021) that result from their vigorous and prolonged endurance training whilst eating a diet restricted in carbohydrates for an extended period; 20 months on average in that trial. The next significant analysis of this phenomenon comes from a series of meticulously conducted studies on Olympic-class athletes (Burke et al., 2017; Burke et al., 2020; Burke, 2021; Burke et al., 2021). Like the results of Volek et al. (2016), these studies reported substantially higher rates of fat oxidation in these elite athletes adapted to the LCHF diet for varying periods. For example, fat oxidation rates measured during exercise at high %VO_2_max were 1.60 g/min at 79%VO_2_max (Burke et al., 2017); 1.25 g/min at 77%VO_2_max (Burke et al., 2020); 1.40 g/min at 74%VO_2_max (Burke et al., 2021). In their most recent publication, these authors (Burke et al., 2021) also reported the time course of this adaptation. Rates of fat oxidation increased from 0.50 g/min to 1.40 g/min in just 5 days following adoption of the LCHF diet. With a return to the habitual HCLF diet, these changes reversed over a similar period. This suggests that this adaptation is not dependent on chronic whole-body training-induced adaptations but is more likely the result of a rapid change (<1 week) in the metabolic and hormonal milieu before and during exercise. The most obvious candidate would be a significant change in blood insulin concentrations and action (glucose-lowering effect of insulin) since even at modest blood concentrations, insulin exerts powerful control over the rates of glucose (Thiebaud et al., 1982) and fat oxidation (Bonadonna et al., 1990; Campbell et al., 1992; Horowitz et al., 1997). In an attempt to understand other factors which influence substrate oxidation during exercise, Rothschild et al. conducted a multivariable regression of 434 studies (Rothschild et al., 2022). Exercise duration, dietary fat intake, age, VO_2_max, and percentage of type 1 muscle fiber all were associated with decreased RER, while dietary carbohydrate intake, exercise intensity, male sex, and carbohydrate intake before and during exercise increased RER. While these factors are important considerations on substrate oxidation and account for 36%–59% of RER variability in these models, it is important to denote that multiple studies have controlled for some, or all, of these variables and still demonstrate robust alteration of substrate oxidation with habituation to LCHF diets when directly assessing these effects in randomized controlled trials (Prins et al., 2023a). Additionally, insulin may also explain why many of these cited factors altered cross-over kinetics (i.e., fitness level/VO_2_max (Lohmann et al., 1978; Wirth et al., 1981; King et al., 1990; Solomon et al., 2015), exercise intensity (Lin et al., 2022), carbohydrate intake (Collier and O’Dea, 1983), FFA availability and oxidation, and enzymatic changes (Puchalska and Crawford, 2017; Puchalska and Crawford, 2021), as discussed below.

Other studies have since reported higher rates of fat oxidation during exercise of moderate to high intensity in athletes adapted over shorter durations to the LCHF diet—1.22 g/min at 71%VO_2_max (Coyle et al., 1985); 1.21 g/min at 72%VO_2_max (Webster et al., 2016); 1.7 g/min at 65%VO_2_max (Webster et al., 2018); 1.7 g/min at 80%VO_2_max (Webster et al., 2018); 0.93 g/min at ∼64%VO_2_max (Rowlands and Hopkins, 2002); 0.78 g/min at 88%VO_2_max (Prins et al., 2019). A value of 0.99 g/min at 70%VO_2_max was measured in subjects who began exercise with reduced muscle glycogen content (Weltan et al., 1998). Even the original study establishing the value of carbohydrate supplementation during prolonged exercise (Coyle et al., 1985) found that athletes who ingested 100 g carbohydrates per hour during prolonged exercise at ∼71%VO_2_max reached fat oxidation rates of >0.95 g/min even without following the LCHF diet. When they ingested a placebo during exercise, their fat oxidation rates reached 1.22 g/min. Another study (Rauch et al., 2022) found that approximately 50% of subjects eating a HCLF diet and ingesting 90 g carbohydrates per hour for the first 2 h of a 3-hour exercise bout at 60%VO_2_max achieved peak fat oxidation rates >1 g/min. While these results demonstrate consistent elevations in fat oxidation on LCHF diets, it is important to contextualize absolute fat oxidation values across studies for protocol differences (i.e., graded exercise, steady state, etc. [Bordenave et al., 2007; Egan et al., 2016)] and the described underestimation of fat oxidation of >70%VO_2_max (Hetlelid et al., 2015). None the less, these findings are incompatible with the cross-over concept.

**FIGURE 3:**
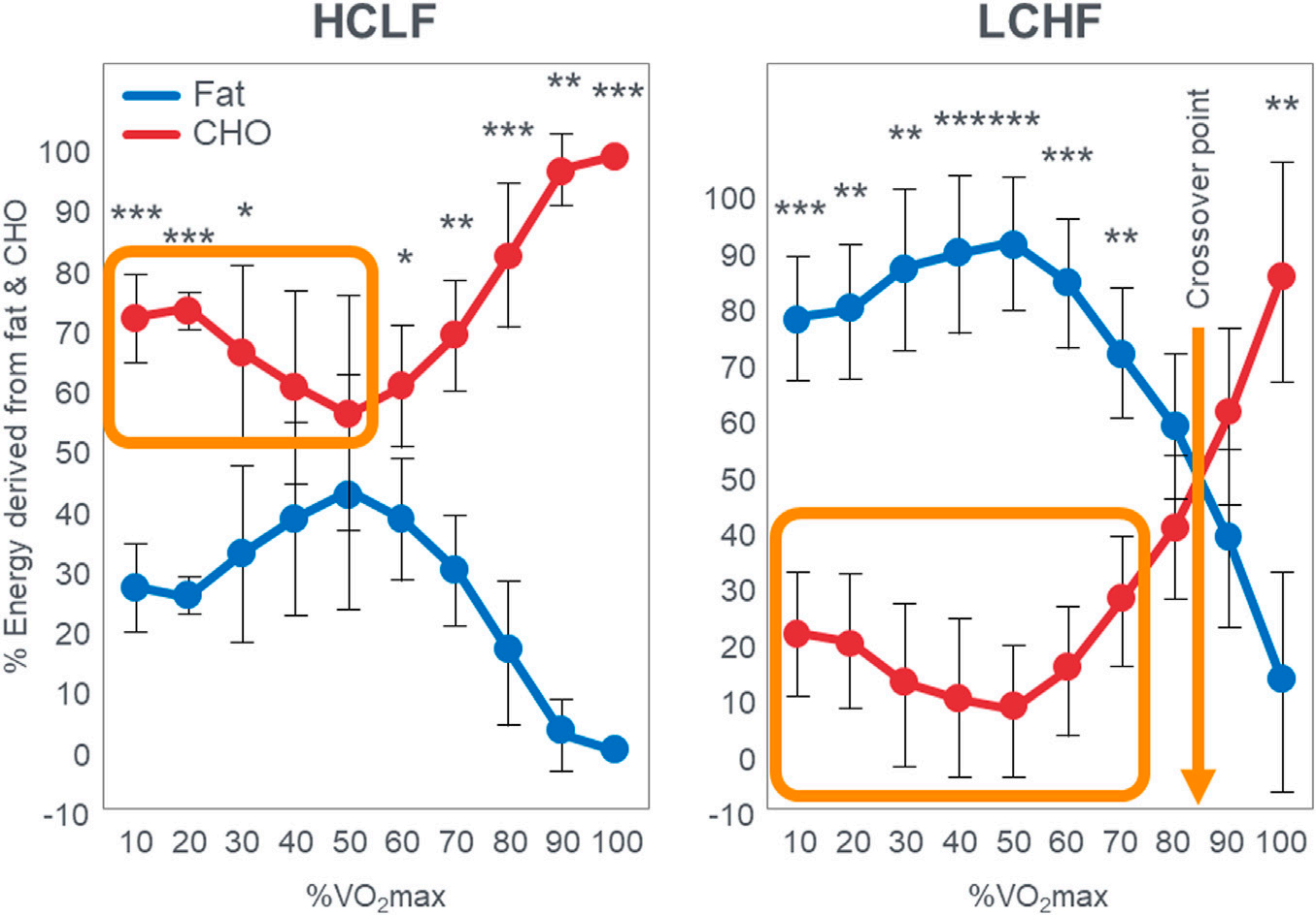
Relative contribution (%) of carbohydrate and fat to energy expenditure during exercise across a range of intensities in response to consuming a HCLF (left panel) or LCHF (right panel) diet for 6 weeks. Left panel; energy expenditure on HCLF diet; Right panel; energy expenditure on LCHF diet, *n* = 7. Data: Mean ± SD. *p < 0.05, **p < 0.01, ***p < 0.001, significant difference between LCHF and HCLF. This is reproduced from reference (Prins et al., 2023b). The orange box in the left panel indicates high rates of carbohydrate oxidation at low exercise intensities with no obvious crossover point when following the HCLF diet. In contrast rates of carbohydrate oxidation are low when following the LCHF diet (orange box in right panel) and there is a clear crossover point at about 85%VO_2_max (arrow). Redrawn from reference (Prins et al., 2023b).

**FIGURE 4:**
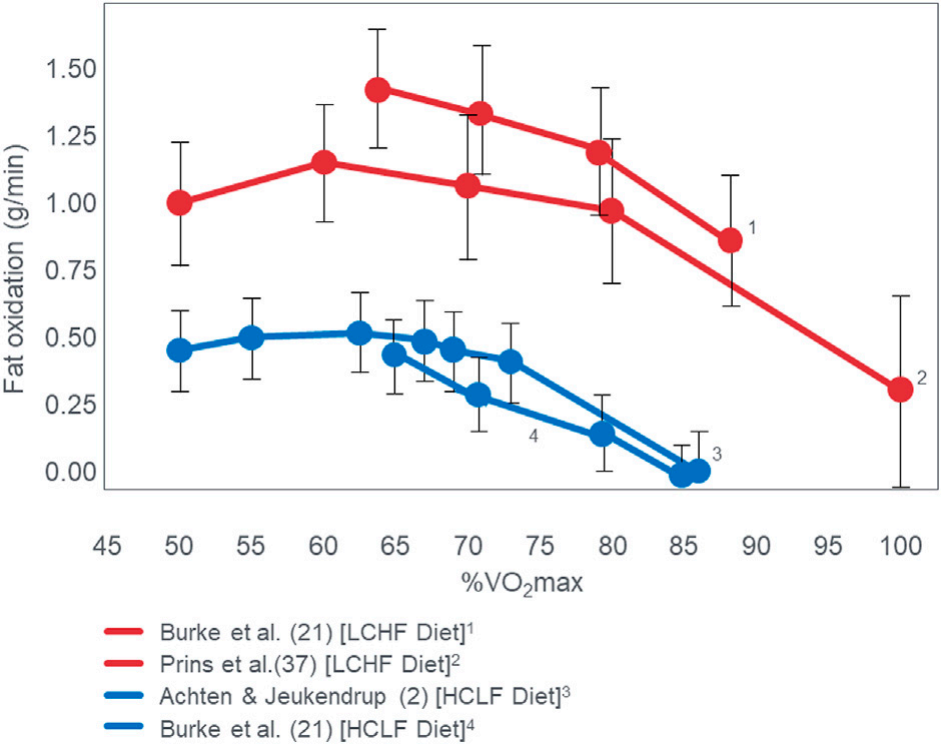
Changes in rates of fat oxidation with increasing exercise intensity (%VO_2_max) in three different studies (Achten and Jeukendrup, 2003; Burke et al., 2021; Prins et al., 2023b). Note the equally high rates of fat oxidation at high exercise intensities in two studies (Burke et al., 2021; Prins et al., 2023b) of athletes quite different in their performance ability, level of training and duration of exposure to the LCHF diet [5 (Burke et al., 2021) vs. 42 (Prins et al., 2023b) days].

Since Prins et al. (2019) performed VO_2_max tests in their subjects when eating either the LCHF or HCLF diets for 6 weeks, they were able to study the effects of this dietary change on the crossover point during progressive exercise to exhaustion [(Prins et al., 2023b); Figure 3]. Interestingly, Figure 3 (left panel) shows that when eating the HCLF diet subjects did not show a definitive crossover point. Instead, they derived more than 50% of their energy from carbohydrate oxidation at all exercise intensities. Peak rates of fat oxidation (0.53 g/min) were achieved at 60%VO_2_max after which values fell progressively, reaching close to zero (0.09 g/min) at 100%VO_2_max. In contrast, when following the LCHF diet, subjects generated 50% or more of their energy requirements from fat even at exercise intensities up to 90%VO_2_max, reaching the crossover point at ∼85%VO_2_max (Figure 3; right panel). When subjects ate the LCHF diet, they showed progressive, exponential changes in carbohydrate oxidation with increasing exercise intensity as predicted in Figure 1. Figure 4 includes the data for peak rates of fat oxidation measured in that study (Prins et al., 2023b) as well as data from Achten and Jeukendrup (Achten and Jeukendrup, 2003) and the most recent study of Burke et al. (Burke et al., 2021). Whereas the data from Prins et al. (2023b) are from well-trained recreational athletes who adapted to the LCHF diet for 6 weeks, the data from Burke et al. (Burke et al., 2021) are from elite Olympic class racewalkers who followed the LCHF diet for just 5 days before resuming their more usual HCLF diet for another 5 days whereafter they were re-tested. These data provide two striking findings. First, amongst the highest rates of fat oxidation (1.5 g/min) yet measured were recorded in the Olympic-class racewalkers following adoption of a LCHF diet for just 5 days (Burke et al., 2017; Burke et al., 2021). These data are very similar to those measured in recreational but well-trained runners who had adapted to the LCHF diet for 6 weeks (Prins et al., 2019; Prins et al., 2023b). While these rapid adaptations may be partially explained by elite-level fitness (Rothschild et al., 2022), recent analyses have demonstrated that respiratory quotient and substrate oxidation changes occur in 4 days in male endurance athletes on an isocaloric LCHF diet (Prins et al., 2019), within 7 days (Hall et al., 2016) and 14 days in moderately trained athletes (Cipryan et al., 2018) and overweight/obese subjects (Buga et al., 2021; Hall et al., 2021), as biochemical changes consistent with increase fat oxidation (i.e., reduced glucose and insulin load; increase ketones) occur upon the first 24 h following reduced carbohydrate intake (<50 g/day) (Hengist et al., 2022). While the timeline for changes and plateaus in substrate oxidation following LCHF adaptations remains unknown, changes are initiated within the first 24 h of LCHF feeding and the plateaus appear to occur at some point within the first two weeks and are likely influenced by the baseline fitness level of each individual. Second, despite this very short adaptation period, rates of fat oxidation over 0.8 g/min were measured even during exercise at 87%VO_2_max in the Olympic racewalkers.

**FIGURE 5:**
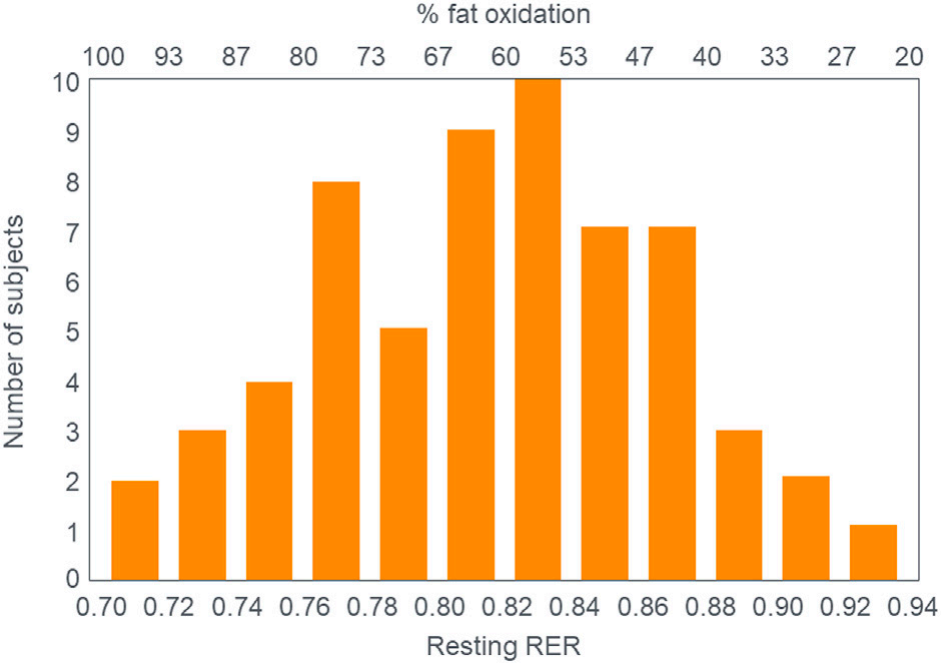
Distribution of Respiratory Exchange Ratios (RER) at rest in 61 subjects in the study of Goedecke et al. (Goedecke et al., 2000). Note that the mean RER at rest was 0.817 indicating that ∼37% of the resting energy expenditure was derived from carbohydrate oxidation. Reproduced from (Goedecke et al., 2000).

**FIGURE 6:**
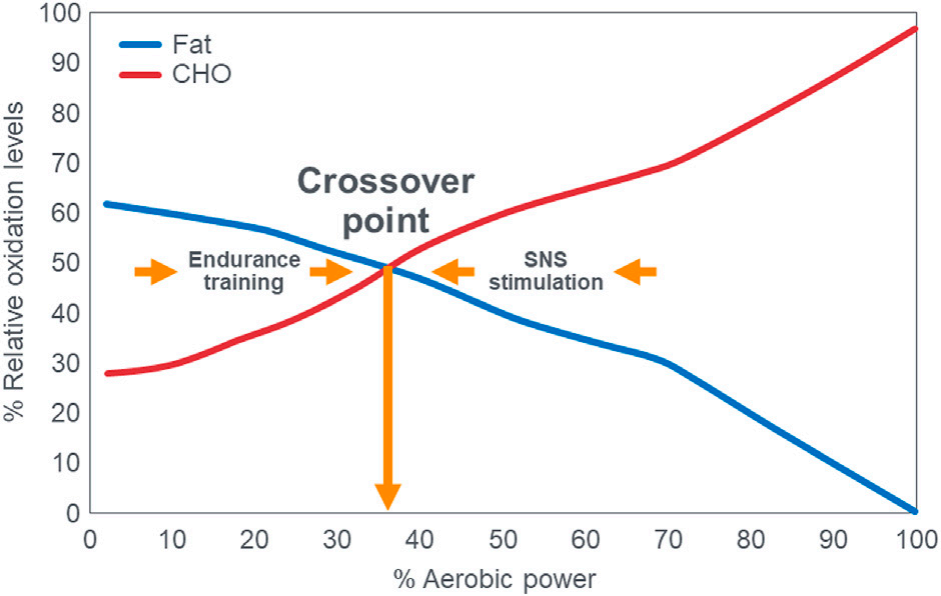
The publication of Kolodziej and O’Halloran (Kolodziej and O’Halloran, 2021) includes this figure which differs substantially from the more usual graph shown in Figure 1. Note that the rate of carbohydrate oxidation of ∼30%–40% at rest and during low-intensity exercise is substantially higher than values in Figure 1. Also, the crossover point occurs at less than 40%VO_2_max. Reproduced from (Kolodziej and O’Halloran, 2021).

These data from two separate research groups therefore unequivocally disprove the popular concept that human athletes are unable to extract any meaningful degree of energy from fat oxidation during exercise at intensities >85%VO_2_max (Figure 2) (Achten et al., 2002; Achten and Jeukendrup, 2003; Venables et al., 1985; Jeukendrup and Wallis, 2005; Randell et al., 2017; Frandsen et al., 2017; Vest et al., 2018; Brooks, 1997; Brooks, 1998; Brooks and Mercier, 1985; Brooks and Trimmer, 1985; Kolodziej and O’Halloran, 2021; Kenney et al., 2021; Ghanassia et al., 2006; Burke, 2015; Hargreaves and Spriet, 2020; Chrzanowski-Smith et al., 2020; Chrzanowski-Smith et al., 2021; Maunder et al., 2018; Hawley and Leckey, 2015; Leckey et al., 1985; Torrens et al., 2016). These data also identify a paradoxical observation included in the original description of the crossover point [ (Brooks and Mercier, 1985)—see earlier] but missing from Figure 1. At low exercise intensities, between 50%–70% of energy was derived from carbohydrate oxidation (outlined in left panel; Figure 3). This conflicts with the graph in Figure 1 but is compatible with findings from an earlier study (Goedecke et al., 2000) showing that the RER at rest is highly variable but is 0.70 (indicating 100% fat oxidation) in less than 5% of subjects (Figure 5). Instead, the mean resting RER in that sample was 0.81 indicating that carbohydrate oxidation provided 37% of the resting energy expenditure in this sample. In a second study from the same laboratory (Goedecke et al., 2001), the resting RER on three separate occasions varied from 0.87–0.88 indicating that 57%–60% of resting energy expenditure was derived from carbohydrate oxidation in those subjects. McNeil et al. (McNeill et al., 1988) reported similar values (0.83) in healthy women; this value increased to 0.86 when subjects increased their dietary carbohydrate content from 44.5% to 54.4% for six days before evaluation. Additionally, 24-hour respiratory quotient data measured in 112 healthy subjects in “energy balance” eating a HCLF diet whilst living in a respiratory chamber for 3 days found that 44% of their energy metabolism at rest was derived from carbohydrates [Table 1 in (Pannacciulli et al., 2007)]. Indeed, the literature includes an alternative figure (Figure 6) of the crossover point which predicts that carbohydrates provide ∼30% of the resting energy expenditure and this rises even during low-intensity exercise, reaching the crossover point at just over 35%VO_2_max (Kolodziej and O’Halloran, 2021).

These findings identify a crucial paradox: If the key (obligatory) role of carbohydrate oxidation during exercise is to provide the energy required for high-intensity exercise (>75%VO_2_max) (Brooks, 1997; Brooks, 1998; Brooks and Mercier, 1985; Brooks and Trimmer, 1985; Kolodziej and O'Halloran, 2021; Kenney et al., 2021; Ghanassia et al., 2006; Burke, 2015; Hargreaves and Spriet, 2020; Chrzanowski-Smith et al., 2020; Chrzanowski-Smith et al., 2021; Maunder et al., 2018; Hawley and Leckey, 2015; Leckey et al., 1985; Torrens et al., 2016), then why does carbohydrate oxidation contribute such a large (∼50%–60%) fraction of the energy expenditure at rest or during low-intensity exercise in some humans, especially if they eat a high-carbohydrate diet (McNeill et al., 1988)? One potential answer may be that carbohydrate oxidation at rest serves a purpose other than energy provision since the oxidation of fat is perfectly capable of covering the energy requirements of humans at rest as clearly shown in the original graph of the crossover point (Brooks and Mercier, 1985; Kenney et al., 2021). Thus, this finding (Figures 3, 6) raises two important questions: First, why is so much energy derived from carbohydrate oxidation at rest or low-intensity exercise if the role of carbohydrates is to provide the obligatory fuel source during high-intensity exercise? Second, why is so much energy derived from fat oxidation during exercise at high intensities (∼85%VO_2_max) on the LCHF diet if fat oxidation is merely a “helper fuel” (Hargreaves and Spriet, 2020) that cannot sustain high-intensity exercise?

### Fat oxidation, mitochondria and insulin

The most recent publication of Prins et al. (2023a) provides more evidence of these paradoxical findings. They studied a group of highly trained recreationally competitive athletes who completed a 1609 m time trial (TT) and subsequently a high-intensity interval running session (6 × 800 m) on a laboratory treadmill following 4 weeks/31 days of adaptation to both the HCLF and LCHF diets. Unexpectedly, subjects exercising at >85%VO_2_max during the 6 × 800 m repetitions when adapted to the LCHF diet achieved the highest average rates of fat oxidation yet measured in humans: peak fat oxidation rates measured at 86.40% ± 6.24% VO_2_max were 1.58 ± 0.33 g/min with 30% of subjects achieving values > 1.85 g/min. Since these athletes were not of Olympic ability but were well-trained middle-aged athletes, this suggests that a key driver of oxidating so much fat and extending the crossover point to ∼85%VO_2_max is probably not superior athletic ability. Although training-induced increases in physical fitness levels are known to lower blood insulin concentrations and increase insulin action in various populations (Borghouts and Keizer, 2000; Roberts et al., 2013; Bird and Hawley, 2016; Thabit and Leelarathna, 2016; Lin et al., 2022), a more likely explanation for these findings is the middle-aged subject’s accumulated mitochondrial volume and capacity through years of endurance training in parallel with the dietary-induced changes on mitochondrial function and blood insulin concentrations. It is known that endurance exercise training increases mitochondrial size, number, and oxidative activity in a dose-dependent manner (i.e., training volume) contributing to improved whole-body glucose metabolism (Holloszy, 1967; Holloszy and Coyle, 1984; Constable et al., 1987; Kim et al., 2008; Bishop et al., 2019). Prins et al. cohort was very fit competitive endurance athletes (VO_2_max: top 5% (Rapp et al., 2018)) with high endurance training volume (running: 50 km/week; 10 years) in their middle age, with considerable capacity to accumulate mitochondrial size and number for maximizing potential substrate oxidation (Prins et al., 2023a). Dietary strategies which dramatically lower insulin (i.e., LCHF/ketogenic diets) have been demonstrated to increase molecular markers of skeletal muscle mitochondrial biogenesis (i.e., PGC-1α; TFAM (Kim et al., 2008; Bishop et al., 2019)) in pre-clinical models over a short duration (3 weeks) independent of exercise (Huang et al., 2021). Additional work has demonstrated that a ketogenic diet may also increase skeletal muscle mitochondrial volume (Parry et al., 2018), and mitochondrial enzymatic activity (Zhou et al., 2021), in preclinical models of aging. Direct ketone administration *via* exogenous ketone diester administration in a preclinical model also improved skeletal muscle regeneration, electron transport chain, and insulin sensitivity (Roberts et al., 2022), suggesting a potential role for ketones in facilitating these changes (Poff et al., 2020). Miller et al. (2020) found that after 12 weeks of a LCHF/ketogenic versus a HCLF diet (both combined with a resistance exercise program), diet did not increase mitochondrial volume. However, the LCHF/ketogenic diet reduced insulin load and insulin resistance, increased whole body fat oxidation, and demonstrated directional improvements in *ex-vivo* mitochondrial ATP production, ATP per Gram of O_2_ consumed, and ATP per Gram of H_2_O_2_ produced from carbohydrates, fat, and ketones when controlling per Gram of skeletal muscle, indicating increased mitochondrial capacity and efficiency in the LCHF/ketogenic diet group relative to the HCLF diet group. Taken together, this data suggests that the record levels of fat oxidation observed in our analyses of middle-aged competitive endurance athletes (Prins et al., 2023a), likely did not result from shifts mitochondrial volume over the 4 weeks LCHF/ketogenic dietary protocol, but instead resulted from mitochondrial function and efficiency changes in the low insulin environment in response to the LCHF/ketogenic diet. The low insulin environment appears to be a critical contributor to these observations as low blood insulin concentrations (Bonadonna et al., 1990; Campbell et al., 1992; Horowitz et al., 1997; Weltan et al., 1998) favor increased adipose tissue lipolysis resulting in increased FFA availability, thereby providing increased substrate in the form of FFA for enhanced skeletal muscle fat metabolism. The opposite is observed in persons with type-2 diabetes whose fitness levels are very low; whose blood glucose and insulin concentrations are high; in whom insulin action is impaired; and who have low rates of fat oxidation, even during low-intensity exercise when fat oxidation should be maximized (Ghanassia et al., 2006). The availability of FFA is a key determinant of fat oxidation and thus ketogenesis, but only in low-insulin environments in which insulin’s direct roles in attenuating both adipocyte lipolysis, hepatic fat oxidation and ketogenesis, and whole-body lipid oxidation (Owen et al., 1969; Balasse, 1979; Moller, 2020; Deru et al., 2021; Hengist et al., 2022) are removed. For example, Hengist et al. (Hengist et al., 2022) have recently reported that a single isocaloric and isoprotein LCHF meal increases fat oxidation and blood ketone concentrations and lowered carbohydrate oxidation post-prandially but only when blood insulin concentrations are very low. Indeed, fat oxidation is exquisitely sensitive to small changes in insulin within the physiological range and the effects are rapid (Bonadonna et al., 1990; Coppack et al., 1994), and result in widespread biochemical changes (Puchalska and Crawford, 2017; Puchalska and Crawford, 2021). Gribok et al., measured 24-h minute-to-minute substrate oxidation *via* whole-body indirect calorimetry and glycemia utilizing continuous glucose monitoring and found excellent agreement between measures of substrate oxidation (RER), glycemia and metabolic changes in response to high and low carbohydrate meals (Gribok et al., 2016) supporting the link between diet, substate oxidation, insulin and glycemia. Thus, accumulated mitochondrial machinery in concert with diet-induced changes in mitochondrial function and insulin load may explain the record levels of fat oxidation observed in Prins et al. middle-aged athletes (Prins et al., 2023a), as well as other observations in long-standing endurance athletes with exceptionally high fat oxidation rates while consuming LCHF diets (Volek et al., 2016; Burke et al., 2021). These shifts in mitochondrial and insulin function may also help explain how fitness level/VO_2_max (Lohmann et al., 1978; Wirth et al., 1981; King et al., 1990; Solomon et al., 2015), exercise intensity (Lin et al., 2022), carbohydrate intake (Collier and O’Dea, 1983), FFA availability and oxidation, and enzymatic changes (Lohmann et al., 1978; Wirth et al., 1981; Puchalska and Crawford, 2017; Puchalska and Crawford, 2021) influence the crossover point, as all these factors influence, or are influenced by, mitochondrial and/or insulin biology, as noted above.

### Glycogen content

Additionally findings from Prins et al., (Prins et al., 2023a), demonstrated that performance during the 1609 mTT was unaffected by the LCHF diet. However, whilst the glycogen content of the leg muscles of recreational athletes eating the LCHF diet may be reduced (Phinney et al., 1983; Webster et al., 2016), they are not zero. Thus, one possible explanation for this unexpected finding could be that recreational athletes eating the LCHF diet still have sufficient muscle glycogen to power one maximal effort lasting ∼6 min. To examine this possibility, the authors included another exercise test involving 6 × 800 m interval running repetitions on a treadmill. This form of repetitive high-intensity exercise rapidly depletes muscle glycogen stores (Impey et al., 2020). Therefore, according to the prevailing hypothesis of an obligatory role for muscle glycogen use during high-intensity exercise (Noakes, 2022), when subjects followed the LCHF diet, muscle glycogen depletion would prematurely limit their exercise performance in the latter stages of the interval session. But the key finding was that exercise performance during the 6 × 800 m repetitions was unaffected by the LCHF diet (LCHF: 1236.1 ± 69.2 s; HCLF: 1254.0 ± 101.2 s). This suggests that despite a predicted greater muscle glycogen depletion following the LCHF diet, athletes performed equally well on either diet. These findings are also in line with recent reviews which show equivalent exercise performance across a range of athletic contexts in subjects when they followed either the LCHF (<50 g carbohydrates/day) or the HCLF diets (McSwiney et al., 2019; Murphy et al., 2021; Noakes, 2022). However, some data suggests that low muscle glycogen may not universally impede high-intensity performance (Vigh-Larsen et al., 2021). However, this appears to be governed by duration of high-intensity exercise and its resulting impact on muscle glycogen content. As an example, one study demonstrated that reduced muscle glycogen content following 3 min of high-intensity exercise did not impede performance (Bangsbo et al., 1992). However, a subsequent high-intensity exercise bout following the initial 3-minute bout did demonstrate reduced exercise performance as a result of greater reductions in muscle glycogen content. Of note, Prins et al. (2023a) demonstrated evidence that 4 weeks habituation to LCHF did not reduce performance even following a repeated sprint protocol which lasted ∼21 min in duration, nor were there decrement in 1609 m time trial performance for exercise lasting between 6–7 min in duration, both of which would be expected to reliably produce pronounced reductions in muscle glycogen content. The reduction in muscle glycogen in Prins et al. (2023a) analyses following 6–21 min of high-intensity exercise is further supported by prior analyses showing that both 4 weeks of eucaloric LCHF feeding and ≥24 weeks LCHF feeding in weight stable athletes resulted in 45%–47% reductions in muscle glycogen at rest (Phinney et al., 1983), and even more pronounced reduction following a single bout of exercise.

## Glucose homeostasis in athletes

### Dysglycemia

**FIGURE 7:**
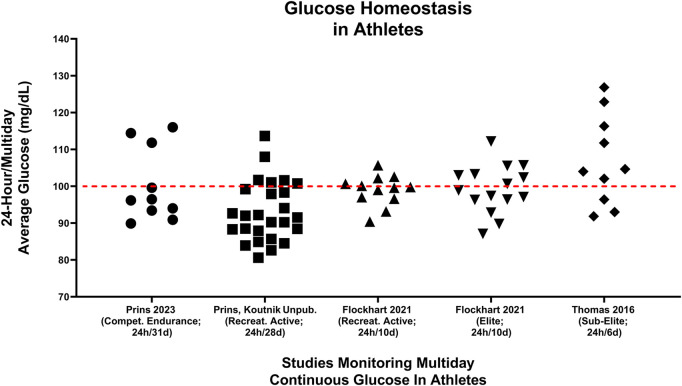
Multiday averages of minute-by-minute continual glucose monitoring from 73 athletes of different fitness calibers are presented across 5 different studies (Thomas et al., 2016; Flockhart et al., 2021; Kulawiec et al., 2021; Prins et al., 2023a) (Prins, Koutnik unpublished). (Thomas et al., 2016) median glucose values were captured cumulative distribution plots of the entire averaged continuous glucose signal over the 6-day period (Figure 5; 0.5; (Thomas et al., 2016)). Prins, Koutnik unpublished data represented continuous glucose values from recreationally athletes over a 4-week duration [cohort: (Buga et al., 2023)]. Median [(Thomas et al., 2016; Prins et al., 2023a) Prins, Koutnik unpublished] or mean (Flockhart et al., 2021) multiday average glucoses values were presented. Red line differentiates individual athlete’s 24 h blood glucose averages above and below 100 mg/dL.

As different diets can produce markedly different metabolic effects, they may also have important impacts on acute- and/or long-term health for athletes. Maffetone and Laursen described the fit but unhealthy athlete in 2015, citing modern-day highly processed, high glycemic diets as a contributing factor (Maffetone and Laursen, 2015). Another unexpected finding from Prins et al. was that 30% (3 of 10) of athletes showed evidence of pre-diabetic blood glucose (100–125 mg/dL) values while eating their usual HCLF diet (Prins et al., 2023a), consistent with pre-diabetes interstitial glucose values using analogous technology (Yost et al., 2020); this phenotype disappeared when they ate a LCHF diet. Importantly, these pre-diabetic glucose values could not be explained by underlying demographics, body composition or physical activity differences as pre-diabetic subjects had near equivalent age (pre-diabetic: 41.7 years/o; cohort: 39.3 years/o), running experience (pre-diabetic: 8.7 years; cohort: 9.7 years), body weight (pre-diabetic: 84.0 kg; cohort: 86.7 kg), BMI (pre-diabetic: 25.4 kg/m^2^; cohort: 26.2 kg/m2), body fat percentage (pre-diabetic: 14.8%; cohort: 15.7%) and VO_2_max (pre-diabetic: 61.0 mL/kg/min; cohort: 58.7 mL/kg/min) when compared to the entire cohort (Prins et al., 2023a). Figure 7 demonstrate 24-hour multiday glycemic averages captured across five studies [(Thomas et al., 2016; Flockhart et al., 2021; Kulawiec et al., 2021; Prins et al., 2023a) (Prins, Koutnik unpublished)]. Thomas et al., (Thomas et al., 2016), and Kulawiec et al. (Kulawiec et al., 2021) reported on the same cohort and found that 30% (3 of 10) of sub-elite normal-weight endurance athletes eating HCLF diets had pre-diabetic fasting glycemic values. Thomas et al., reported an even higher percentage of athletes >100 mg/dL (7 of 10) when assessing the entire 24-hour/6 days free-living glycemic profile. Flockhart et al. (2021) found that 30% (4 of 12) of recreationally active athletes and 47% (7 of 15) of competitive endurance athletes (i.e., elite) had 24-hour mean glucose values > 100 mg/dL over a two-week period ((Flockhart et al., 2021); author correspondence). Our team found that a smaller percentage of (12%; 3 of 26) recreationally active college students ≤23 years of age presented with >100 mg/dL over a 4-week HCLF observational period (Prins, Koutnik unpublished). These preliminary observations suggest that a small percentage of athletes may be developing features of pre-diabetes while consuming HCLF diets.

Potential limitations of these assessments include acute exercise-induce hyperglycemia, intensified training mitochondrial dysfunction, method of capture, differences between fasting, post-prandial and 24-hour glucose, and glycemic bias (improvements) from continuous glucose monitoring devices. Acute exercise training (Ishihara et al., 2020; Prins et al., 2023a) has been demonstrated to elevate glucose values during exercise. Multiweek intensified training has also been demonstrated to induce short-term mitochondrial and glycemic dysfunction (Flockhart et al., 2021). However, exercise typically makes up ≤ 17% of total daily hours even in elite athletes (Fiskerstrand and Seiler, 2004) and results in increased insulin sensitivity for up to 16 h afterwards (Borghouts and Keizer, 2000) attenuating the prospect that acute hyperglycaemia during exercise is driving these elevation in 24-hour glucose values. In an initial controlled analysis of a progressive intensified training program, mitochondrial dysfunction, and acute signs of dysglycemia were discovered (Flockhart et al., 2021). However, Thomas et al. (2016), Kulawiec et al., (2021), and Prins et al. (2023a), all captured free-living and fasting windows and Prins et al. (2023a), specifically controlled for activity throughout the entire 4-week study period to ensure training volume was consistent, suggesting intensified training cannot explain the elevation in fasting and 24-h glucose values. Importantly, these glycemic phenotypes were captured utilizing 24-hour, minute-by-minute continuous glucose monitoring as it tracks long-term to HbA_1c_ (Bergenstal et al., 2018; Hirsch et al., 2019; Valenzano et al., 2021), short term continuous glucose monitoring readings (10–14 days) are good estimates of 3-month continuous glucose monitoring averages (Chehregosha et al., 2019), and emergent evidence suggest that continuous glucose monitoring metrics track better with mean glucose (r = 0.92–0.98) than HbA_1c_ (r = 0.71) in larger trails (Beck et al., 2019). Post-prandial eating windows on HCLF diet can lead to elevated glucose values during non-fasted window and influence 24-hour/multiday glycemic values. However, these pre-diabetic glycemic values in a percentage of athletes following standard HCLF dietary approaches [(Prins et al., 2023a), (Thomas et al., 2016; Flockhart et al., 2021; Kulawiec et al., 2021)] were not just during post-prandial/feeding windows but represented 24-hour mean glucose values across multiple days. Prins et al. (2023a), Thomas et al. (2016), and Kulawiec et al. (2021) all reported >100 mg/dL fasting values in 30% of athletes which attenuates the possibility that these pre-diabetic glycemic values are just a consequence of post-prandial glucose elevations. None the less, these findings in athletes were observed despite the known bias for improved glycemia in the general population when utilizing glycaemic monitoring tools and enrolled in research studies (Poolsup et al., 2013; Bailey et al., 2016; Liao and Schembre, 2018; FORTMANN et al., 2020; Liao et al., 2020; Yost et al., 2020; Dehghani Zahedani et al., 2021; Dicembrini et al., 2021; Whelan et al., 2021; Chekima et al., 2022; Naguib et al., 2022). Together, these data suggest that acute exercise-induce hyperglycemia, intensified training mitochondrial dysfunction and method and timing of capture cannot negate these pre-diabetic glycemic findings.

In line with these observation in athletes engaging in regular exercise, Dehghani Zahedani et al. (2021) also reported that many people who self-classified as healthy were reclassified as pre-diabetic or diabetic upon further analyses of continuous glucose values of ≥10 days. While the exact consequences of these pre-diabetic glucose values in populations regularly engaging in physical activity requires additional analyses, and additional confirmation is warranted, it appears unlikely that persistent and elevated glucose values would be recommended granted the demonstrated adverse effects of poor glycemic control is dose-dependent, accumulate over the lifespan, and are not completely reversible despite future glycemic improvement (Diabetes et al., 1993; Holman et al., 2008; Forbes and Cooper, 2013; Nathan et al., 2013; Lind et al., 2014; Nathan and Group, 2014; Nordwall et al., 2015; Diabetes, 2016a; Diabetes, 2016b; Diabetes, 2016c; Stahl et al., 2017; Lind et al., 2019; El Malahi et al., 2022). Together these data preliminarily suggest that even young to middle age, very physically active (i.e., endurance athletes), normal bodyweight individuals may develop signs of undetected pre-diabetic glucose values which may be attributed to dietary choice and Prins et al. (Prins et al., 2023a) found that all subjects resolved their pre-diabetic glycemic phenotype when following a LCHF/ketogenic diet without reported changes in calories consumed or in exercise training load, or as a result of differences in body composition across groups. This appears to directly implicate diet as a modifiable factor in glycemic control, even in seemingly “healthy” athletes who develop pre-diabetic glucose values without risk factors or overt diagnoses of carbohydrate intolerance. Interestingly, individuals with the highest 4-week average blood glucose concentrations while eating their usual HCLF diet also experienced the greatest glycemic benefits when switching to the LCHF diet. These improvements in glycemic control were also associated with very high levels of fat oxidation measured during exercise, clearly demonstrating key links between HCLF glycemic homeostasis, diet-induced changes in glycemic control, and diet-induced changes in rates of fat oxidation.

### Dysglycemia, mitochondria and insulin

Mitochondrial and insulin biology may partially, or completely, explain these observations (Thomas et al., 2016; Flockhart et al., 2021; Prins et al., 2023a) of pre-diabetic glycemic values in athletes consuming a HCLF diet. First, it is established that mitochondrial volume, function, and adaptation decline with age (Trounce et al., 1989; Cooper et al., 1992; Boffoli et al., 1994; Short et al., 2005; Menshikova et al., 2006; Kim et al., 2008) and mitochondrial dysfunction is associated with insulin resistance and load through reduced fat-oxidation, increased reactive oxygen species, and reduced ATP production, which is not completely explained by changes in mitochondrial volume (Kim et al., 2008). Second, skeletal muscle is integral to the development of insulin resistance and is a major reservoir for postprandial glucose storage (Shulman et al., 1990; Meyer et al., 2002; Karlsson et al., 2006; Pagel-Langenickel et al., 2010). In skeletal muscle, disruption of mitochondrial biology is evident in some insulin-resistant subjects years before they develop diabetes (Patti et al., 2003; Petersen et al., 2004; Befroy et al., 2007) and continuous glucose monitoring has been utilized to observe mitochondrial dysfunction through dynamic and continuous glycemic monitoring (Flockhart et al., 2021). These combined observations help explains our findings which demonstrate that middle-aged athletes with robust mitochondrial machinery and without overt pathology may have developed reduced mitochondrial function leading to pre-diabetic glycemic values observed *via* continuous glucose monitoring (Prins et al., 2023a). This is supported by other analyses also demonstrating pre-diabetic glycemic values in a similar percentage of athletes, of which 2/3 were middle-aged (Thomas et al., 2016). Importantly, Prins et al., demonstrated that a LCHF/ketogenic diet reversed this pre-diabetic glycemic phenotype and improved glycemic control in virtually all subjects, independent of changes in calories, activity, or body composition (Prins et al., 2023a), suggesting diet plays a central role in regulating this phenotype. Metabolic dysfunction in concert with insufficient insulin action might explain worsened glycemic control on the HCLF diet whereas reduced blood insulin concentrations as a byproduct of lower blood glucose concentrations on the LCHF diet will facilitate higher rates of fat oxidation and improved glycemic control. In line with our observations, individuals with untreated pre-diabetes (HbA1c: 6.2%) were able to reduce HbA1  c by 0.23%–0.26% and lower fasting blood glucose concentrations by 7.0–10.3 mg/dL, simply by reducing carbohydrate intake to less than 100g/day without fully achieving a “ketogenic” phenotype (<50 g carbohydrates/day with elevation in resting blood beta-hydroxybutyrate concentrations). This confirms that the LCHF diet is a viable treatment option for improving pre-diabetic glucose control (Dorans et al., 2022). Interestingly, the greater reductions in mean blood glucose concentrations (14.9 mg/dL) in our study (Prins et al., 2023a) compared to those achieved in a recently reported randomized controlled trial (7.0 mg/dL) (Dorans et al., 2022), may reflect the greater restriction in carbohydrate consumption in our study (40.9 g carbohydrates/day) compared to those in the study of Dorans et al. (96 g carbohydrates/day). When considering the known link between mitochondria and insulin function (Kim et al., 2008; Pagel-Langenickel et al., 2010), and the demonstration that LCHF/ketogenic diets improve insulin sensitivity, and mitochondrial molecular signaling and function (Newman et al., 2017; Parry et al., 2018; Miller et al., 2020; Huang et al., 2021; Zhou et al., 2021; Roberts et al., 2022), the established reductions in the blood insulin concentration when switching from a HCLF to a LCHF diet, may explain how these relatively rapid changes between diet, insulin, glucose homeostasis, and fat oxidation might be linked.

## Conclusion

In summary, the evidence presented here shows that when adapted to the LCHF diet athletes burn more fat at much higher exercise intensities than is predicted by the crossover concept, and athletes burn more carbohydrates during low-intensity exercise than is allowed by the crossover concept which theorizes that carbohydrate contribution to energy production at low exercise intensities is very low since carbohydrate is the obligatory fuel for high-intensity exercise. Of note, recent findings also suggest that LCHF is not an inherently inferior nutritional approach for physical performance and LCHF diets may have important health implications, particularly improved glycemic control, even for athletes engaging in regular exercise. The diet-induced shift in the crossover point produced by the LCHF diet may be explained by the dietary-induced changes on mitochondrial function, lower blood insulin concentrations and the resulting biochemical changes which allow augmented adipose tissue lipolysis and whole-body rates of fat oxidation across a wide range of exercise intensities without marked decrement in performance. The crossover curve cannot explain the high carbohydrate oxidation at low exercise intensities since that curve predicts very low or minimal carbohydrate contribution to fuel oxidation during low-intensity exercise. But we and others have observed substantial amounts of carbohydrate oxidation at rest (Figure 5) and during low-intensity exercise (Figure 3; left panel). This requires a different explanation and should encourage a reconsideration of the true biological basis for the crossover curve.

In his 1971 Banting Memorial Lecture entitled Physiology of Insulin in Man, George Cahill wrote: “The *Rules of the game.* Mammals and particularly man, appear to abide by several general metabolic guidelines relating to fuel homeostasis. These rules, for those so interested, provide fertile ground for teleologic speculation since they have or must have had major survival value. One of these is to maintain glucose levels within very narrow limits, returning the level rapidly to the norm if perturbed in either direction” [(Cahill, 1971), page 785]. In 2009, Wasserman further emphasized the biological priority of glucose homeostasis stating (Wasserman, 2009), “Four grams of glucose circulates in the blood of a person weighing 70 kg … a sophisticated control system is in place to maintain blood glucose constant … The essential role of glucagon and insulin and the importance of distributed control of glucose … ” Cahill recognized three mechanisms by which an elevated blood glucose concentration can be returned “rapidly to the norm if perturbed”. The first is increased blood glucose oxidation. This is achieved by insulin-induced inhibition of lipolysis thereby increasing carbohydrate in place of fat oxidation. The second is to store glucose as glycogen either in the liver or in the skeletal muscles. And the third is to convert any remaining excess glucose to fat (Cahill, 1971). Wasserman also emphasized these points but went on to describe the influence of blood flow, capillary recruitment, spatial barriers at the extracellular levels, transport number and activity at the cellular membrane, and enzymatic capacity, activity, and spatial barriers at the intracellular level all as additional regulatory mechanisms (Wasserman, 2009). This raises the possibility that perhaps an important role for carbohydrate oxidation in modern humans, particularly those who are largely physically inactive, might be to aid in the regulation of blood glucose concentration especially when they eat their usual HCLF diets. In support of this was the linear association between the change in glycemic control and rates of fat oxidation with the two different diets [Figure 8 in (Prins et al., 2023a)]. Since the evidence is inconclusive (Prins et al., 2019; Noakes, 2022; Prins et al., 2023a; Prins et al., 2023b) that muscle glycogen is the key “obligatory” energy fuel for all forms of high-intensity exercise, as is generally believed (Leckey et al., 1985; Burke, 2015; Hawley and Leckey, 2015; Torrens et al., 2016; Hargreaves and Spriet, 2020; Noakes, 2022), we offer alternative speculation: A key reason for the rapid muscle glycogen storage in athletes exposed to the HCLF diet (Ivy et al., 1985) may be to assist in the more efficient homeostatic regulation of their blood glucose concentrations. Our finding that 30% of otherwise healthy lean middle-aged athletic individuals, show evidence for impaired 24-hour glucose tolerance (pre-diabetes) when eating the HCLF diet (McNeill et al., 1988), a finding supported by others (Maffetone and Laursen, 2015; Thomas et al., 2016; Flockhart et al., 2021), with complete resolution on the LCHF diet (McNeill et al., 1988), suggests that the repeated, persistent challenge posed by the HCLF diet to Cahill’s *First Rule of the Game,* may ultimately lead to a failure of glucose homeostasis regulatory systems in some, potentially explained through mitochondrial dysfunction and subsequent initiation of insulin resistance, placing individuals at risk for future metabolic disease and related complications (Reaven, 1988).
